# Supplementary material for: Blood CD8+ Naïve T-Cells Identify MS Patients with High Probability of Optimal Cellular Response to SARS-CoV-2 Vaccine
Source: Vaccines (Basel). 2023 Aug 22;11(9):1399. doi: 10.3390/vaccines11091399 (PMC10535818; doi:10.3390/vaccines11091399)
Supplement: Supplementary file 1 [file vaccines-11-01399-s001.zip › vaccines-2538642-supplementary.pdf]

vaccines

MDPI

Article

Blood CD8+ Naïve T-Cells Identify MS Patients with High Probability of Optimal Cellular Response to SARS-CoV-2 Vaccine.

SUPPLEMENTARY MATERIAL

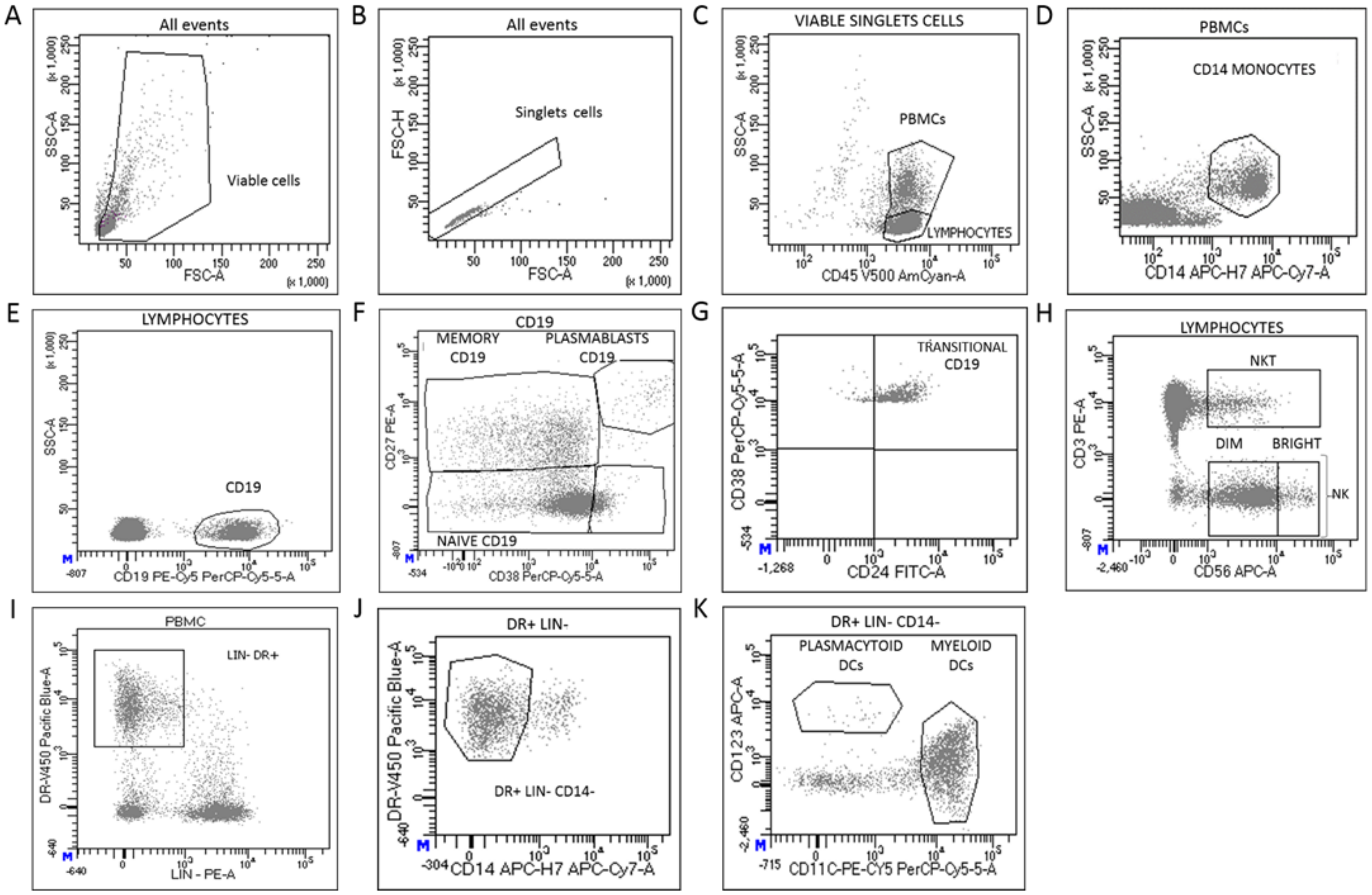

Figure S1. Gating strategy for the identification of Monocytes, B Cells, CD56+ cells and Dendritic cells subsets. Total events were first gated to exclude apoptotic cells and debris (A) and then gated for doublet discrimination (B). Cells were further analyzed to identify peripheral blood mononuclear cells (PBMCs) (C), including lymphocytes, which were identified based on CD45 staining. Monocytes were defined as CD14+ cells (D). Among lymphocytes, the expression of CD19 allowed us to identify B cells (E). B cells were divided into four types based on the expression of CD38 and CD27 antigens in: naïve (CD19+ CD38dim CD27-), memory B cells (CD19+ CD27dim CD38dim), plasmablasts (CD19+ CD27hi CD38hi) (F), and transitional B cells (CD19+ CD27- CD24hi CD38hi) (G). Additionally, within the lymphocyte subset, the expression of CD3 and CD56 was used to identify Natural Killer T-cells (CD56+, CD3+), Natural Killers dim cells (CD3- CD56+/low), and Natural Killer bright cells (CD3- CD56high) (H). Dendritic cells were also identified as HLA-DR+ CD3- CD19- CD56- cells (I), along with CD14- cells (J). Within this population, myeloid (CD123-/low CD11c+) and plasmacytoid (CD123+ CD11c-/low) subtypes were identified (K).

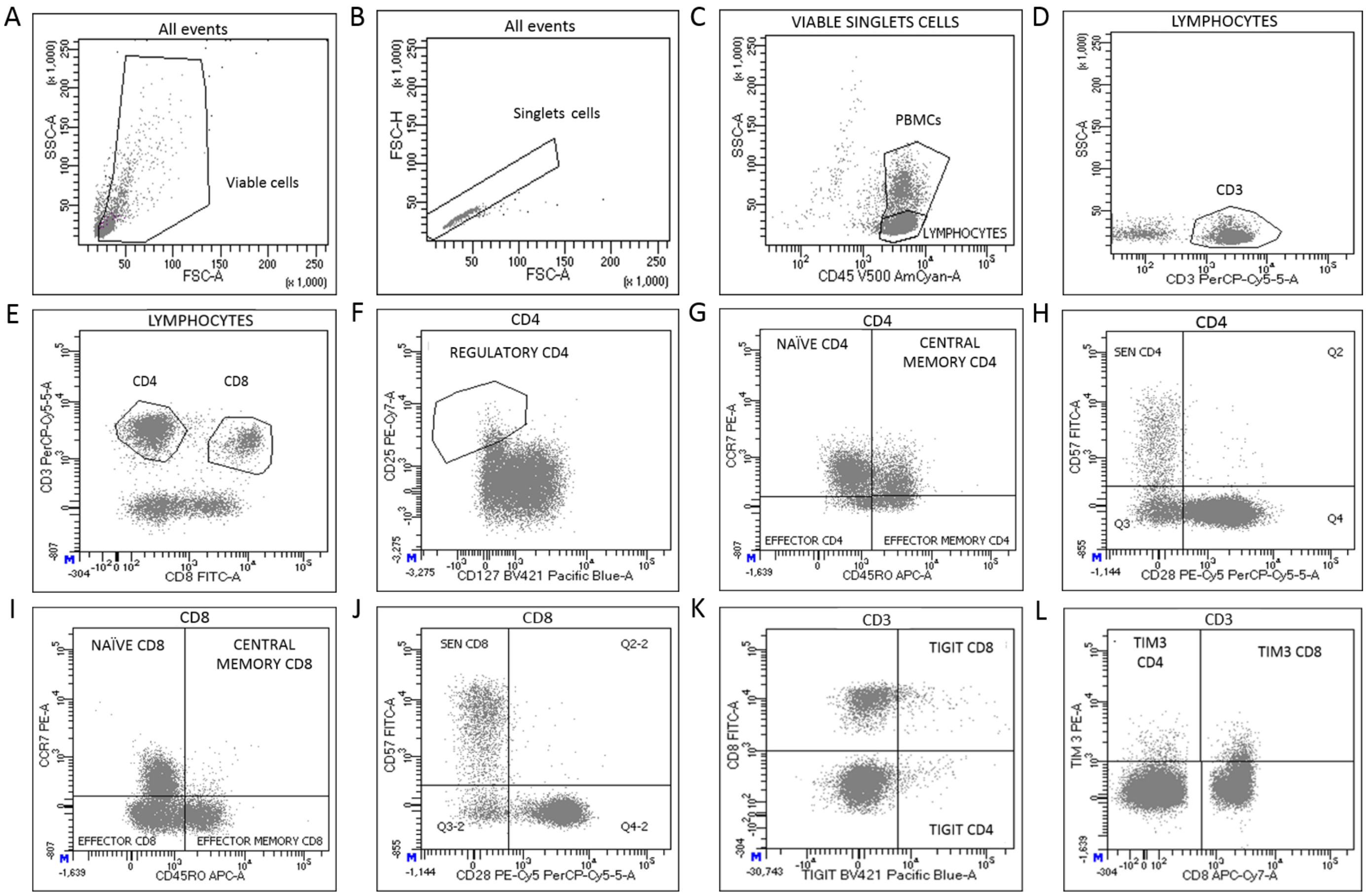

Figure S2. Gating Strategy of T-cells. Selection of viable cells based on forward scatter (FSC) and side scatter (SSC) characteristics (A). Doublets were excluded from the analysis (B). CD45+ cells were gated (C). T-cells were identified as CD45+ CD3+ cells based on their expression of CD3 (D). Helper and cytotoxic T-cells were gated within the T-cell population based on the expression of CD4+ and CD8+, respectively (E). Regulatory T-cells were defined as CD3+ CD4+ CD25high CD127-/low cells (F). CD4+ T-cell subpopulations were characterized as naïve (CCR7+ CD45RO-), central memory (CCR7+ CD45RO+), effector memory (CCR7- CD45RO+), and terminally differentiated (CCR7- CD45RO-) (G). CD4+ senescent cells were identified as CD3+ CD8- CD57+ CD28- cells (H). CD8+ T-cells were also categorized into naïve, central memory, effector memory, and terminally differentiated subpopulations based on the expression of CCR7 and CD45RO (I). CD8+ senescent cells were identified as CD3+ CD8+ CD57+ CD28- cells (J). The expression of TIGIT (K) and TIM3 (L) was observed within both CD4+ and CD8+ T-cell populations.
